# Hepatocellular Carcinoma with Prominent Intracytoplasmic Inclusions: A Report of Two Cases

**DOI:** 10.1155/2016/2032714

**Published:** 2016-10-12

**Authors:** Adeline R. Chelliah, Jasim M. Radhi

**Affiliations:** ^1^Department of Pathology, Cork University Hospital, Cork, Ireland; ^2^Laboratory Medicine, Peterborough Regional Health Centre, Peterborough, ON, Canada

## Abstract

Hepatocellular carcinoma (HCC) is the commonest primary malignant neoplasm of the liver in most countries with a notoriously poor prognosis. Variation in global incidence is well-recognized and the occurrence of HCC is linked to several established environmental, dietary, and lifestyle factors. HCC demonstrates morphological heterogeneity both within the same tumor and from patient to patient. Differing architectural patterns and cytological variants may be seen. Inclusion bodies are believed to represent organized structures of proteins which contribute to their pathogenesis and share several constituents like chaperones, p62, ubiquitin, and Valosin containing protein. The various hepatocyte cytoplasmic inclusions described in HCC include Mallory-Denk bodies (MDBs), hyaline bodies (HBs), glycogen, fat, fibrinogen, alpha 1 antitrypsin (AAT), and ground glass. MDBs are the most common inclusions seen in hepatocellular carcinomas. The two cases shared intracytoplasmic inclusions which are characterized by larger sizes and present in every section examined. These exhibited features of MDBs and HBs present in most tumor cells, further supporting close relationship.

## 1. Case Histories

The first patient is an 83-year-old man who presented with vague upper abdominal discomfort, weight loss, and general weakness. Blood work was normal and upper and lower gastroscopy revealed no specific lesion or malignancy.

A solitary lesion in the left lobe of liver was found on computed tomography (CT) scan. Tumor markers were noncontributory and hepatitis serology was negative.

He has a medical history of hypertension controlled with antihypertensive medications. Liver biopsy showed hepatocellular carcinoma. Surgical resection of segments 2 and 3 including the mass was performed.

The second patient is a 56-year-old female who presented to the emergency department with abdominal pain. Medication used included oral contraceptive and hormonal supplements. CT scan showed 11 cm heterogeneous liver mass in the right lobe with evidence of intralesional hemorrhage and associated contrast extravasation requiring urgent surgical intervention. The lesion was resected. Hepatitis serology result was negative.

**Figure 1:**
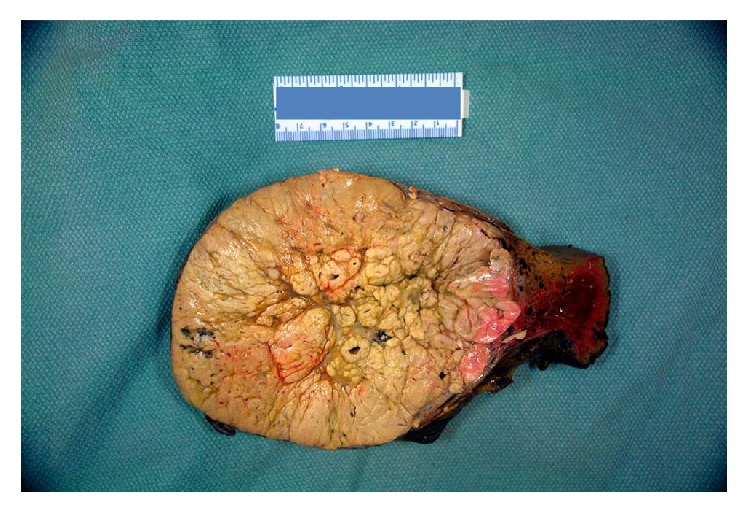
Resected hepatocellular carcinoma.

Macroscopic examination of the first lesion showed a partial hepatectomy with 4 cm lobulated green/yellowish mass with surrounding nodular and fibrotic liver parenchyma. The second lesion consisted of an ill-defined tumor, 13 cm size, with a yellow/brown cut-surface and a small peripheral rim of grossly normal liver parenchyma (Figure 1).

**Figure 2:**
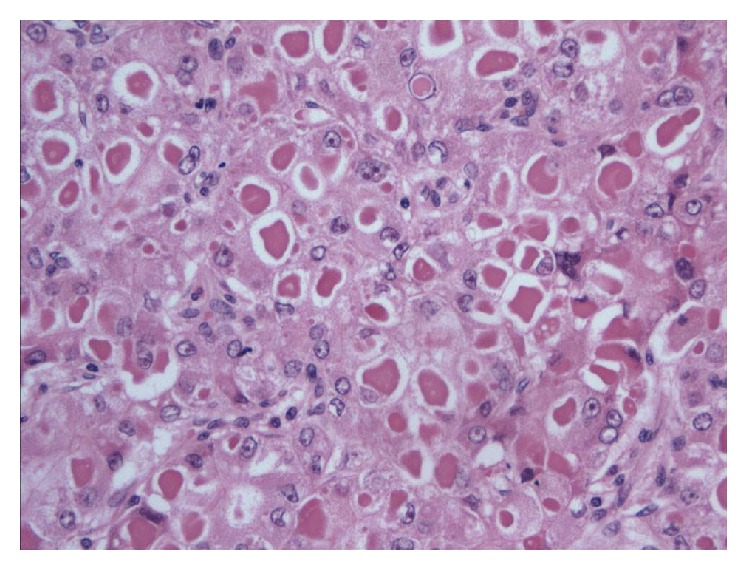
Eosinophilic cytoplasmic inclusions with globular appearance and surrounding clear halo.

**Figure 3:**
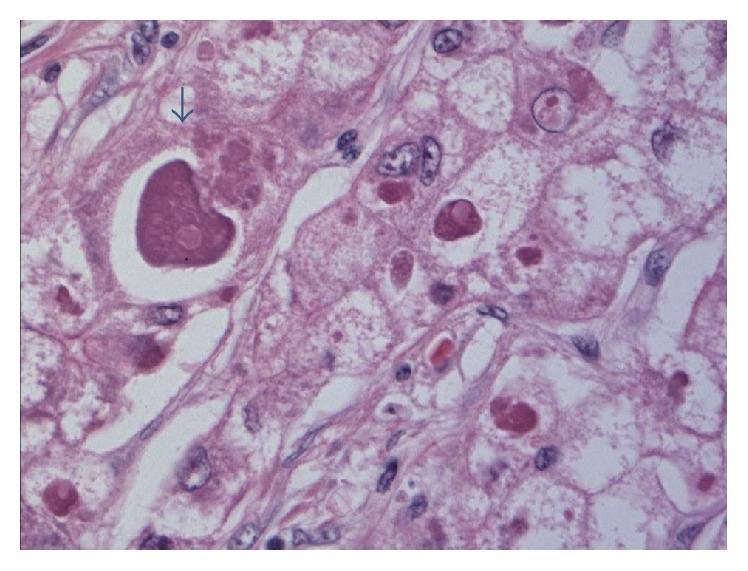
This photograph shows hyaline and Mallory-Denk bodies within the same inclusion (arrow).

Microscopically, both cases showed HCC with admixed trabecular and pseudoacinar patterns. The malignant hepatocytes were enlarged and contained prominent, densely eosinophilic intracytoplasmic globules, many of which had surrounding clear halos admixed with Mallory hyaline bodies (arrow) (Figures 2 and 3).

The globular structures were negative for Masson's trichrome, amyloid, and Periodic Acid Schiff/Periodic Acid Schiff Diastase (PAS/PASD) special stains.

**Figure 4:**
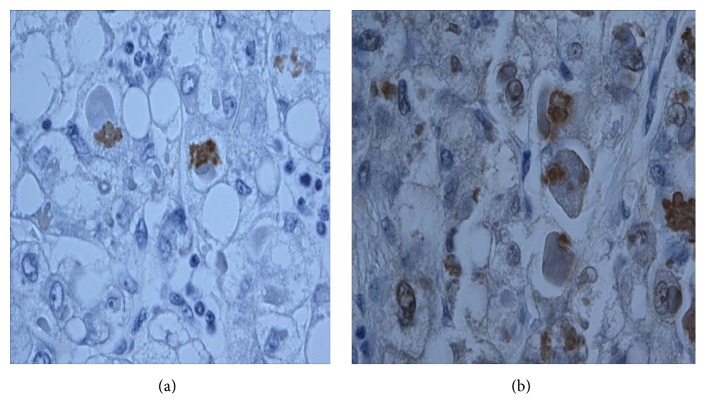
(a) The inclusions demonstrate focal ubiquitin expression by immunohistochemistry. (b) The cytoplasmic inclusions are positive for p62 immunomarker.

By immunohistochemistry (Dako, Carpinteria, CA, USA) the intracytoplasmic globules showed expression of p62 and focal positivity for ubiquitin (Figures 4(a) and 4(b)).

CAM5.2 showed very focal, weak expression. Fibrinogen, alpha-fetoprotein, AAT, alpha 1-antichymotrypsin, and fibrinogen immunohistochemistry were all negative. The morphology and predominant CAM5.2 negativity in the majority of the inclusions suggest they are mainly HBs while the focal, weak CAM5.2 staining indicates a focal MDB component.

Case one had occasional neoplastic hepatocytes that also contained cytoplasmic fat and was associated with changes of nonalcoholic steatosis in the adjacent liver.

The background hepatic parenchyma in case two demonstrated changes consistent with cirrhosis.

Electron microscopy revealed dense fibrillary material arranged in parallel with occasional vesicular structures present.

## 2. Discussion

Hepatocellular carcinoma (HCC) is the commonest primary liver cancer in most countries with a recognized global variation in incidence, being most prevalent in China, Southeastern Asia, and Sub-Saharan Africa [1]. There is a higher incidence in men, in whom it is the fifth most frequently diagnosed cancer worldwide but the second most common cause of cancer-related death. Prognosis remains poor with most studies reporting 5-year survival rates of <5% in symptomatic patients [1].

Chronic viral infection (HBV or HCV), alcohol induced liver injury, ingestion of high levels of aflatoxin, iron overload (hereditary hemochromatosis), and alpha 1 antitrypsin deficiency are amongst the accepted risk factors for HCC. The tumor commonly arises in cirrhotic livers.

Symptoms may be due to the tumor or the predisposing chronic liver disease and include abdominal pain, general fatigue, anorexia, weight loss, nausea, or vomiting.

Microscopically, HCCs show loss of the normal reticulin network with widening of the cell plates (>3 cells thick). The tumor cells resemble hepatocytes to a variable extent, depending on the degree of differentiation. The presence of intercellular bile and identification of canaliculi confirm the hepatic cell lineage. They may show small or large cell change and be associated with cytological atypia, mitotic activity, and “capillarisation” of the sinusoids.

The report described two cases which presented to the emergency with abdominal pain. On CT scanning both patients showed single hepatic mass. No past history of liver diseases was noted and serology testing for viral hepatitis was negative. The initial clinical diagnosis for the first case was metastatic carcinoma and for the second case it was hepatic adenoma. Following proper clinical assessment both patients underwent surgical resection of the masses which revealed HCC with florid intracytoplasmic inclusions.

Intracytoplasmic inclusions are not uncommon or specific to HCC. MDBs, first described by Mallory in 1911 [2], are the most well-recognized and have since been reported in a range of nonneoplastic and neoplastic processes, including alcoholic and nonalcoholic steatohepatitis, Wilson's disease and other copper accumulation disorders, cholestatic conditions such as primary biliary cirrhosis (PBC), Indian childhood cirrhosis, exposure to drugs such as amiodarone, focal nodular hyperplasia, hepatic adenoma, and HCC. MDBs occur in approximately 20–30% of HCCs while HBs are seen in about 20% of cases. Apart from MDBs, other hepatocyte cytoplasmic accumulations described include HBs, AAT globules, glycogen (clear cells), fibrinogen (pale bodies), ground glass inclusions, fat, and other plasma proteins. P62 is prevalent in inclusions and most implicated in aggregate formation and its overexpression enhanced MDB production. HBs are eosinophilic round or ovoid globules of variable sizes. The exact chemical composition is still unknown but p62 is considered one of the prominent components of this cytoplasmic inclusion [3].

Denk et al. [4] demonstrated a close relationship between MDBs and HBs, both of which are aggregates of misfolded proteins forming as a result of hepatocellular stress. Keratins 8 and 18, ubiquitin, and p62, a stress-inducible ubiquitin-binding protein, are key components of MDBs. While HBs also contain p62 as a major constituent [5], they are distinct from MDBs in that they lack keratin. MDBs appear as irregular, eosinophilic, rope-like aggregates in the cytoplasm while HBs are well-circumscribed, eosinophilic globules surrounded by a clear halo. MDBs express keratins 8 and 18 (granular pattern), p62, and ubiquitin by immunohistochemistry. In contrast, HBs are keratin negative although they do express p62. Ubiquitin is seen in some but not all HBs. MDBs and HBs may sometimes be present in the same cell, forming hybrid inclusions [4].

The inclusions vary in sizes and can be few or numerous with zonal distribution. Special histochemical and immunohistochemical markers are required to assist in delineating some of the other inclusions such as AAT globules and fibrinogen.

Many of the hepatocyte intracytoplasmic inclusions are not specific for any disease entity but identifying a particular type of inclusions in the appropriate histological and clinical setting can point to a particular diagnosis.

The prognosis of HCC is a controversial [6] issue; however, the presences of single lesions which are amenable to surgical treatment, and low grade tumors showing no vascular invasion or metastases. In addition the stable underlying liver diseases in both cases suggest a long survival. The presence or absence of hepatic inclusions or tumor differentiation has not been established as prognostic indicators.

## 3. Conclusion

We report two cases of HCC with unusually florid large, prominent intracytoplasmic inclusions with features of HBs and MDB present. This is in support that both inclusions share common molecular constituents and pathogenesis.
